# Reactive Infiltration and Microstructural Characteristics of Sn-V Active Solder Alloys on Porous Graphite

**DOI:** 10.3390/ma13071532

**Published:** 2020-03-27

**Authors:** Yubin Zhang, Xinjiang Liao, Qiaoli Lin, Dekui Mu, Jing Lu, Hui Huang, Han Huang

**Affiliations:** 1 Institute of Manufacturing Engineering, Huaqiao University, Xiamen 361021, China; 15734003989@163.com (Y.Z.); xinjiangliao@sina.cn (X.L.); lujing26@hqu.edu.cn (J.L.); huangh@hqu.edu.cn (H.H.); 2 School of Materials Science and Engineering, Lanzhou University of Technology, Lanzhou 730000, China; linqiaoli@foxmial.com; 3 School of Mechanical and Mining Engineering, The University of Queensland, QLD 4072, Australia; han.huang@uq.edu.au

**Keywords:** reactive wetting, infiltration, mass transfer, porous graphite, Sn-V alloy

## Abstract

In this work, the reactive wetting and infiltration behaviors of a newly designed Sn-V binary alloy were comprehensively explored on porous graphite for the first time. It was discovered that 0.5 wt.% addition of V can obviously improve the wettability of liquid Sn on porous graphite and the nominal V contents in Sn-V binary alloys has minor effects on the apparent contact angles wetted at 950 °C. Moreover, the V-containing Sn-V alloys were initiated to spread on porous graphite at ~650 °C and reached a quasi-equilibrium state at ~900 °C. Spreading kinetics of Sn-3V alloy on porous graphite well fitted in the classic product reaction controlled (PRC) model. However, our microstructural characterization demonstrated that, besides vanadium carbide formation, the adsorption of V element at the wetting three-phase contact line spontaneously contributed to the reactive spreading and infiltrating of Sn-V alloys on porous graphite. Meanwhile, the formation of continuous vanadium carbides could completely block the infiltration of Sn-V active solder alloy in porous graphite. Affected by the growth kinetics of vanadium carbides, the infiltration depth of Sn-V alloys in porous graphite decreased at increased isothermal wetting temperatures. This work is believed to provide implicative notions on the fabrication of graphite related materials and devices using novel V-containing bonding alloys.

## 1. Introduction

Graphite has a high thermal conductivity, low coefficient of thermal expansion (CTE), and superior wear resistance that makes it widely applied as enhancing particles in metal matrix composites (MMCs) and as a protective coating. Moreover, owing to its excellent lubricating properties, high abrasion, and thermal shock resistance, graphite was often jointed to metals to achieve unique functionality for applications in automotive and nuclear industries. To ensure the satisfying connection between graphite and metals, at least two major issues should be well addressed: (1) poor wettability of liquid metals on graphite; (2) substantial thermal residual stresses caused by the mismatch between the CTEs of metals and graphite. For instance, Chu et al., Sung and Sung reported that improper wetting could result in voids formation at the metal/graphite interface, which led to a dramatic and undesirable loss of thermal conductivities of sintered MMCs [1], or the reliability of brazed graphite joints [2]. To date, surface metallization and active brazing using filler alloys that contain carbide-forming elements (e.g., Ti, Cr, V) were the two key methods to improve the wettability of graphite. Casalegno et al. metalized W, Mo, and Cr on C/C composite and reported the resultant interfacial carbides can significantly improve the wettability of Cu on C/C composite, thus enhancing the bonding strength [3]. Xiong et al. designed a Cu-Au-Pd-V filler alloy that allows the diffusion of V element to C_f_/SiC surface to form VC_0.75_ carbides in the brazed C_f_/SiC-C_f_/SiC joints, and reported have a three-point bend strength of 135 MPa at room temperature [4]. In particular, due to its simplicity, excellent cost-effectiveness, and high bonding strength, the active brazing technique has recently received wide research attention. Morscher et al. fabricated C/C and carbon foam/Ti composites using Ag-32.25Cu-1.75Ti (in wt.%) filler alloy [5]. Recently, Zhang et al. fabricated graphite/Cu joints by Ni-12.6Cr-9P-10Cu (in wt.%) filler alloy at 900–980 °C and revealed that the mechanical strength of the joints was influenced not only by the interfacial products formed between graphite and Ni-Cr-P alloy filler alloy, but also by the residual thermal stress in the graphite joints brazed at high temperatures [6]. Park et al. stated that, given a certain mismatch of CTEs, the residual thermal stress in ceramics/metals joints is directly related to the brazing temperature [7]. These results well agreed with Zhong et al.’s work (2009) that the cracks or fracture mainly occurred through the interfacial beam between the graphite and filler alloys [8]. To alleviate the residual thermal stress, Qin and Feng introduced Cu and Mo as soft interlayers to increase the wettability and improve the mechanical properties of brazed TC4-C/C composite joints [9]. In addition, adding or in situ synthesizing particles with low CTEs in the brazing filler alloys are recognized as a potential method. Song et al. used the graphene nanoplatelets as a reinforcement phase to reduce the CTE of filler alloy and alleviate the residual stresses at the interface of brazed C/C composite [10]. Lin et al. in situ synthesized TiB whiskers during the brazing of C/C composites and TiB_w_/Ti6Al4V composite by Cu-Ni/Ti_2_B filler alloy, in order to lower the residual thermal stress of brazed joints [11]. However, Wang et al. pointed out that challenges remain to evenly distribute these transit phases with minimum defects in the brazed seam [12]. Considering the thermal residual stress is directly related to brazing temperature, Yu et al. [13] and Tsao et al. [14] applied active filler alloys of lower fusibility to braze Al to graphite or Al-graphite composite, respectively; however, ultrasonic vibration or external pressure was needed to assist the wetting of bonding metals on graphite.

Although graphite has been bonded to metals at a wide range of process temperatures from 250 °C to over 1000 °C, existing studies were mainly concerned with the isothermal spreading of Ti/Cr-containing alloys on graphite, and the isothermal wetting temperatures were significantly higher than the conventional process temperatures of graphite joints or MMCs. For instance, Mortimer and Nicholas studied the wetting behaviors of Cu-Cr and Cu-V alloys on HX30 graphite and vitreous carbon at 1145 °C, and reported Cr can improve wettability both substrates and V only improved wettability of Cu on vitreous carbon [15]. Devincent and Michal investigated the wettability of Cu-Cr alloys on graphite using the sessile drop method at 1130 °C, and indicated that reactive spreading is limited by the Cr diffusion to the moving three-phase contact line [16]. Yang et al. revealed that the wettability of Cu-Ti alloys on porous graphite at 1100 °C was closely related to the Ti content and the equilibrium contact angles were dominated by the nature of the reaction layer TiC [17]. Mao et al. reported that the wetting spreading of Cu-Ti alloys on graphite at 1100 °C was affected by the contact area between the liquid phase of Cu-Ti alloy and the graphite substrate, as well as the actual contents of Ti in the liquid phase of Cu-Ti alloys [18]. Yang et al. investigated the spreading kinetics of Cu-Cr alloys on graphite at 1100 °C and stated that the spreading kinetics was initially chemical reaction controlled and then limited by the diffusion of Cr to the advancing triple line [19]. Because previous studies were carried out at temperatures above 1000 °C (1100–1150 °C), their inference to minimize the thermal residual stress of graphited joints, and more scientifically important, to explore the wetting mechanism of bonding metals on graphite at relatively lower temperatures, were limited. Recently, Fu et al. studied the wetting behaviors and bonding of graphite by Sn-0.3Ag-0.7Cu-Ti/Cr (in wt.%) alloys and indicated that the Ti- and Cr-containing Sn-based alloys initiated to wet graphite at 600 and 750 °C, respectively [20]. Furthermore, Fu et al. revealed that the spreading kinetics of Sn-0.3Ag-0.7Cu-3Ti (in wt.%) on graphite at 900 to 1050 °C could be described by the chemical reaction controlled model [21]. Our previous studies on the wettability of Sn-Cr [22] and Sn-Ti [23] alloys on chemical vapour deposition (CVD) diamond clearly indicate that the initiation wetting temperature is well below the brazing temperatures using conventional filler alloys and hence, provided a solid foundation for the development of brazing techniques, i.e., designing Cu-Sn-Cr filler alloy that can considerably remained the mechanical integrity of brazed diamond grits [24]. Previous studies clearly showed that V is one of the carbide-forming elements. Xiong et al. reported the fabrication of C_f_/SiC composites using Pd-Co-V [25] and Cu-Au-Pd-V [4] filler alloys, respectively. Yamazaki and Michal studied the interface reaction between diamond and Ag-Cu-V brazing-filler metal, and proposed the small mismatch in the unit cells of vanadium carbides and diamond may lessen the residual stress [26]. However, little was known about the reaction wetting mechanism of V-containing alloys on graphite, as well as the growth behaviors of vanadium carbide on wetted graphite.

In this work, wetting and infiltration characteristics of Sn-V active solder alloys on porous graphite substrate were systematically studied. The apparent contact angles variation was in situ recorded, and the reactive spreading kinetics of Sn-V alloys on porous graphite was studied at different wetting temperatures. The wetting mechanism was elucidated through a comparative analysis on the spreading kinetics of Sn-3V molten droplets on porous graphite, associated with microstructural characterization at three-phase contact line wetted after different thermal histories.

## 2. Experimental Details

### 2.1. Materials

**Figure 1:**
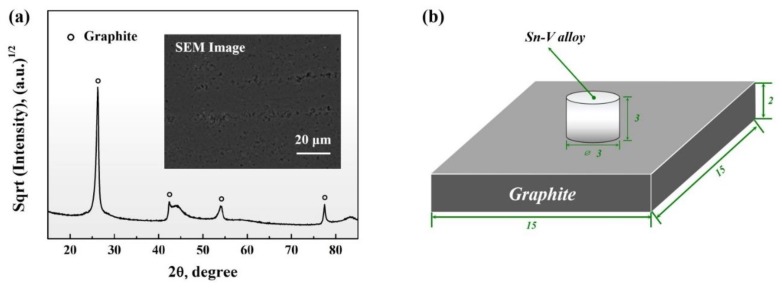
(**a**) XRD pattern and SEM image of prepared porous graphite substrates; (**b**) Schematic illustration of sample assembling.

High purity (99.99 wt.%, Xiamen Tungsten Co. Ltd., Xiamen, China) graphite blocks with a porosity of approximately 18–20 vol.% were cut into substrates of dimensions of 15 mm wide and 3 mm thick using a Struers Minitom low-speed diamond machine (Struers Co. Ltd., Ballerup, Denmark). The substrates were then polished using waterproof silicon carbide sandpapers of up to 2000 mesh in sequence. The polished graphite surface had an average surface roughness of 0.055 ± 0.026 µm, being measured at nine random locations. Figure 1a shows the XRD spectrum of the polished graphite and its typical surface topography. Sn-*x*V (*x* = 0, 0.5, 1, 3, 5, 7, in wt.% unless mentioned elsewhere) powder alloys were prepared by mechanically mixing, which was carried out using weighted amount of Sn (purity of 99.99 wt.%, average grain size of 48 µm) and V (purity of 99.99 wt.%, average grain size of 40 µm) powders (Changsha Tianjiu Co., Ltd., Changsha, China) in a universal 3-dimensional mixer for 3 h to ensure a homogenous composition. Around 0.12 g mixed powder was mechanically pressed into cylindrical ingots in stainless steel die with a diameter of 3 mm at 400 MPa. Prior to sample assembling, all experimental species were cleaned in an ultrasonic alcohol bath.

### 2.2. Wetting Experiment

As demonstrated in Figure 1b, wetting samples were assembled by placing the Sn-V solder alloy ingot in the center of the porous graphite substrate. The wetting was carried out in a quartz chamber using the sessile drop method under a vacuum of below 1 × 10^−3^ Pa. During the vaccum pumping process, the air in the pores of the graphite substrate was also extracted and would have a negiligible effects on wetting behaviour of Sn-V alloy. For the wetting at continuously increased temperatures, the assembled samples were first heated to 350 °C at a rate of 30 °C/min and maintained for 30 min for thermal homogeneity. The wetting temperature was then raised to 950 °C at a constant heating rate of 2 °C/min. Isothermal wetting was carried out by heating the samples to peak temperatures of 750, 800, 850 and 900 °C at a rate of 30 °C/min and held until the apparent contact angle became stable. All wetted samples were cooled to ambient temperature in the quartz tube furnace. The projective profiles of Sn-*x*V alloys were in situ collected using a single lens reflect camera (Nikon D5200, Nikon Corp., Tokyo, Japan), and the apparent contact angle (*θ*) and base radius (*R*) were computed by use of a Drop-analysis software with an accuracy of ±2° for apparent contact angles and ±2% for radius, respectively.

### 2.3. Microstructural Characterization

Selected wetted samples were sectioned perpendicularly to the wetting interface, mounted in epoxy resin, and then mechanically polished for microstructural characterization. A field emission SEM (SIGMA 500, Carl Zeiss AG, Jena, Germany) equipped with an energy dispersive spectrometer (EDS) was used. To investigate the interfacial reaction products, some of the samples were etched in a 30 vol.% HCl aqueous solution to remove the remaining Sn-V alloys. The exposed reaction interface was examined using an X-ray diffractometer (X’Pert pro, PANalytical B.V., Almelo, Holland), and field emission transmission electron microscope (TEM, Talos F200X, Thermo Fisher Scientific Inc., Waltham, MA, USA). Note that the Pt was introduced as a protective coating during TEM sample preparation.

## 3. Results and Discussion

### 3.1. Spreading Characteristics

**Figure 2:**
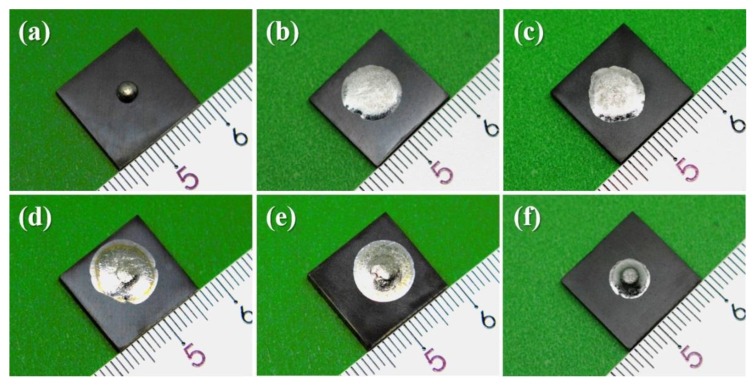
Wetted Samples cooled from 950 °C: (**a**) Sn, (**b**) Sn-0.5V, (**c**) Sn-1V, (**d**) Sn-3V, (**e**) Sn-5V, and (**f**) Sn-7V (in wt.%).

Figure 2 presents the illustrative images of the solidified samples after wetting at the temperature of 950 °C. The high apparent contact angle as shown in Figure 2a indicated the poor wettability and chemical inertness of pure Sn on porous graphite. Doping of 0.5–1 wt.% V to Sn obviously enhanced the wettability of liquid Sn on porous graphite substrates, as shown in Figure 2b,c. As the V content increased to 3 wt.%, a small flat platform was observed on the top of Sn-3V droplet, as given in Figure 2d. When V contents of Sn-V alloys were further increased to 5 and 7 wt.%, flat platforms became more visible on the top of solidified Sn-5/7V alloys, as shown in Figure 2e,f, where the spreading area of Sn-7V alloy decreased significantly.

**Figure 3:**
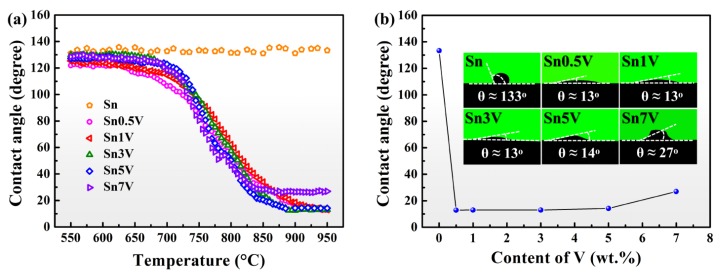
(**a**) Plotting of apparent contact angles of Sn-*x*V (*x* =0, 0.5, 1, 3, 5, 7, in wt.%) alloys versus continuous increase of wetting temperatures; (**b**) Plotting of final apparent contact angles against nominal V contents and corresponding images of intersections of the samples at 950 °C.

Figure 3a shows the apparent contact angles varied with the increased temperature for Sn-*x*V (*x* = 0, 0.5, 1, 3, 5, 7) alloys. The apparent contact angle of liquid Sn on porous graphite substrate fluctuates slightly around 133°, indicating that liquid Sn preserved its chemical inertness on porous graphite substrate at temperature up to 950 °C. However, the trace addition of active V element into Sn could lead to an excellent wetting of liquid Sn on porous graphite, as all of the V-containing Sn-V alloys started to spread on the porous graphite substrate at ~650 °C. Below 900 °C, the apparent contact angles of the alloy decreased with the increase of wetting temperature. When the wetting temperature was above 900 °C, the Sn-V active solder alloy reached a quasi-equilibrium stage. As shown in Figure 3b, the final apparent contact angles remained almost constant after wetting at 950 °C, and corresponding projective images suggested the V contents in Sn-V alloys slightly influenced the final apparent contact angles. When the content of V was 0.5–5 wt.%, the final apparent contact angles were stable at 13°, indicating that trace doping of V element can ensure an excellent wetting of Sn-V binary alloy on porous graphite. The final apparent contact angle conversely decreased to ~27° when V content increased to 7 wt.%. According to our previous study [27], the solubility of V in liquid Sn was ~0.72 at.%, which was much higher than the critical V content of vanadium carbides at 950 °C. The 0.5 wt.% (1.17 at.%) V addition into Sn was therefore sufficient to support the formation of interface vanadium carbides. Hence, the nominal V contents in this work have a minor influence on the final apparent contact angles of Sn-V alloys on porous graphite. The cross-sectional microstructures of the solidified samples wetted at 950 °C given in Figure S1 in supplementary materials and our previous studies revealing the extensive concentration of Sn-Ti IMCs formed through the peritectic reaction on CVD diamond [23] and sapphire mono-crystal [28] indicated that the occurrence of the flat platform shown in Figure 2d–f was likely due to the formation of refractory Sn-V intermetallics in the molten alloy droplets, which impeded the spreading of liquid Sn on porous graphite.

**Figure 4:**
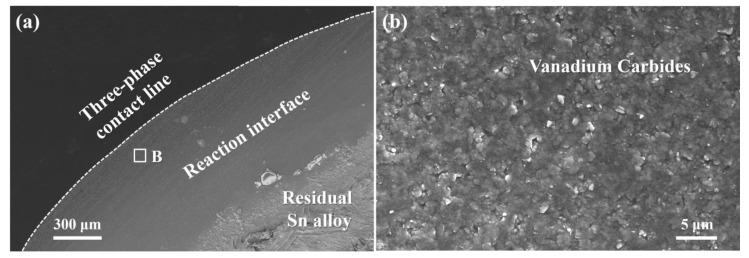
(**a**) Near three-phase contact line morphology between Sn-3V alloy and porous graphite after wetting at 950 °C; (**b**) surface morphology manifested from the location marked by rectangle B in (**a**) after the remained Sn-3V alloy was partly etched by HCl solution.

Figure 4a presents low-magnification SEM micrographs of the interfacial microstructures between Sn-3V alloy and porous graphite after wetting at constantly increased temperature up to 950 °C. The solidified Sn-V alloy droplet was partially etched in an HCL solution and a reaction layer could be observed at the three-phase contact line as shown in Figure 4b, in which a continuous and dense layer of vanadium carbides was recognised by the EDS analysis. EDS analysis of the cross-sectional reaction layer (see Figure S2 in supplementary materials) and the XRD patterns acquired at the reaction interface between Sn-V alloys and porous graphite substrates after wetting at 950 °C (see Figure S3 in supplementary materials) indicated that the vanadium carbides were composed of V_2_C, V_8_C_5_ or V_6_C_5_. According to the work reported by Gremillard et al. [29], reactive spreading requires the adsorption of active elements to the three-phase contact line and the successive occurrence of interface reaction between the active element and the substrate material. However, according to the work reported by Eustathopoulos et al. [30], Saiz and Tomsia [31], whether the spreading kinetics in a reactive wetting system was controlled by the adsorption or the reaction product at the three-phase contact line remains unclear.

**Figure 5:**
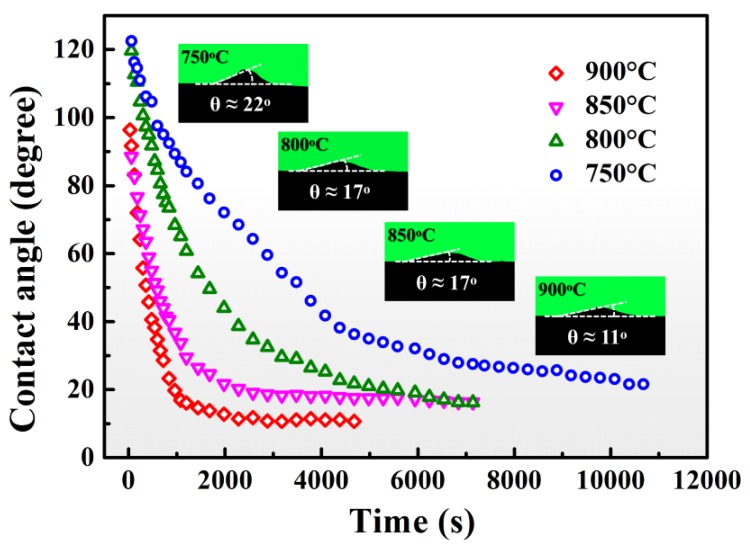
Time sequence of the apparent contact angles of the Sn-3V alloy on porous graphite isothermally wetting at 750, 800, 850, and 900 °C. The inserts correspond projective images of isothermally wetted samples.

To study the spreading kinetics of Sn-3V alloy on porous graphite, isothermal wetting was carried out at 750, 800, 850, and 900 °C. Figure 5 shows the variation of the apparent contact angle of Sn-3V alloy on porous graphite during isothermal wetting. The equilibrium contact angles were 22°, 17°, 17°, and 11° at 750, 800, 850, and 900 °C, respectively. Furthermore, the time to reach the equilibrium state for liquid Sn-3V alloy droplets was approximately 10,380, 5280, 2600, and 2100 s at 750, 800, 850, and 900 °C, respectively. Hence, the equilibrium contact angles and spreading kinetics of Sn-3V alloy on porous graphite were influenced by wetting temperatures.

**Figure 6:**
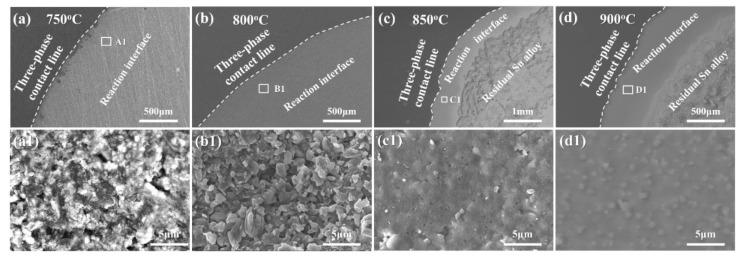
Surface morphologies at the wetting three-phase contact line between Sn-3V alloy and porous graphite after isothermal wetting at (**a**) 750 °C, (**b**) 800 °C, (**c**) 850 °C and (**d**) 900 °C; (**a1**), (**b1**), (**c1**), and (**d1**) were obtained at locations marked as rectangles A1 in (**a**), B1 marked (**b**), C1 marked in (**c**) and D1 marked in (**d**), respectively.

Figure 6a–d show the overall surface morphologies near the three-phase contact line of the Sn-3V alloys on the porous graphite after isothermal wetting at 750, 800, 850, and 900 °C, respectively. Figure 6a1,b1 are the magnified SEM images from the area marked by the white box in Figure 6a,b, showing that after isothermal wetting at 750 and 800 °C the structures of vanadium carbides formed at the three-phase contact line were discontinuous. A continuous and dense layer of vanadium carbides were formed at the three-phase contact line at wetting temperatures of 850 and 900 °C, as shown in Figure 6c1,d1. Clearly, the growth kinetics of vanadium carbides at the three-phase contact line was dependent on wetting temperature.

**Figure 7:**
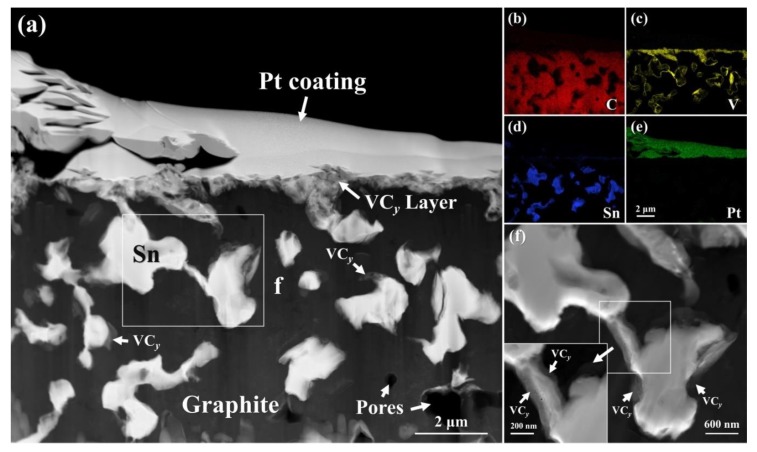
(**a**) cross-sectional HAADF TEM image obtained at the three-phase contact line between Sn-3V alloy and porous graphite after isothermal wetting at 850 °C and corresponding elemental distribution of (**b**) C, (**c**) Sn, (**d**) V, and (**e**) Pt; (**f**) was acquired at the location marked as rectangle f in (**a**). Note that the partial Sn at the three-phase contact line was removed and Pt was introduced as a protective coating.

TEM characterization was performed to acquire detailed microstructures at the wetting three-phase contact line. Figure 7a shows the high angle annular dark field (HAADF) TEM image obtained at the wetted edge of the Sn-3V alloy after isothermal wetting at 850 °C on porous graphite. Figure 7b–e shows the TEM EDS mapping of C, V, Sn, and Pt elements, respectively. Apparently, the horizontal spreading process of the Sn-3V alloy on porous graphite was accompanied by vertical infiltration of liquid Sn into porous graphite. The analysis of the contrast of HADDF images in Figure 7a,c also indicated that continuous precipitation of vanadium carbides at the triple line between liquid Sn and porous graphite, which was consistent with the growth morphologies of vanadium carbides at 850 °C (see Figure 6c1). Meanwhile, the vertical infiltration process was also companied with the formation of vanadium carbides at the liquid Sn/porous graphite interface at the edges of the pores in the porous graphite (see Figure 7a,c,f). To further reveal the composition of the vanadium carbides layer, the XRD patterns were acquired at the reaction interface after wetting at 750, 800, 850, and 900 °C, as shown in Figure S4 in supplementary materials. The XRD patterns and EDS analysis in the cross-sectional reaction interface indicated that the vanadium carbides are V_2_C, V_8_C_7_ or V_6_C_5_.

For reactive wetting systems, the wetting spreading is typically controlled by either the diffusion of reactant to the advancing three-phase contact line, or the chemical kinetics of reaction occurring at the three-phase contact line, and both cases belong to the well-known product reaction controlled (PRC) model. Correspondingly, the diffusion-limited and chemical reaction-limited models of the spreading kinetics were proposed by Mortensen et al. [32] and Dezellus et al. [33], respectively. The diffusion-limited spreading model, in which the spreading rate of the liquid droplet follows a linear relationship with the dynamic contact angle, can be simplified as [19]

(1) $$\frac{dR}{dt}\;=\;C\;\left(\theta -\theta_{e}\right)$$

where *R*, *t*, *θ*, *θ_e_*, C stands for the drop base radius, isothermal dwell time, dynamic contact angle, equilibrium contact angle, and constant for a given system, respectively. The chemical reaction-limited spreading model can be described as [33]

**Figure 8:**
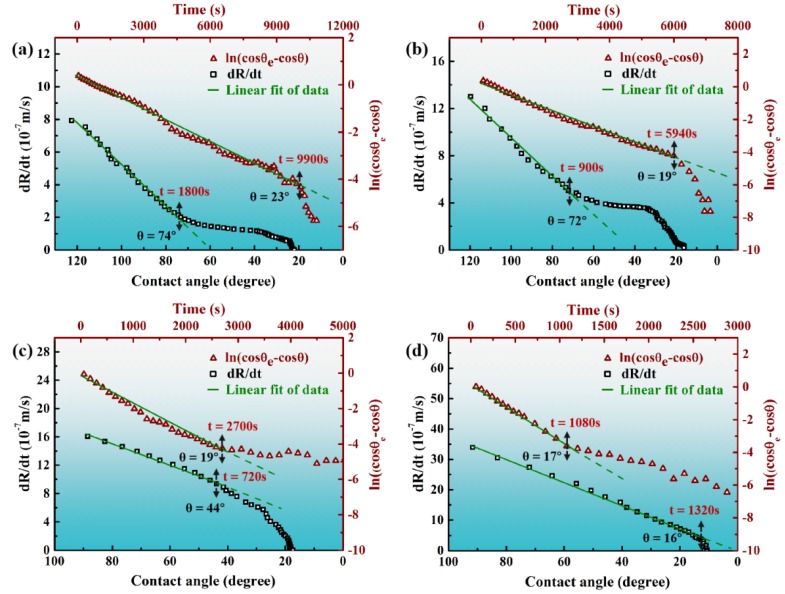
Plots of spreading rate (*dR/dt*) against contact angles and *ln(cosθ_e_ − cosθ)* values of Sn-3V alloy on porous graphite against isothermal dwell time at (**a**) 750 °C, (**b**) 800 °C, (**c**) 850 °C, and (**d**) 900 °C.

*ln(cosθ_e_ − cosθ) = ln(cosθ_e_ − cosθ_0_) − kt* (2)

where *θ_0_*, *θ*, *θ_e_*, corresponds to the initial, dynamic, and equilibrium contact angle, respectively, *t* is the dwell time, and *k* is a kinetic constant depending on the activity of reactive solute and the wetting temperature. The spreading characteristics of Sn-3V alloy on porous graphite can be comparatively examined using Equation. (1) and (2). As shown in Figure 8a–d, the values of *ln(cosθ_e_ - cosθ)* are entirely linearly proportional to the isothermal dwell time of Sn-V alloy at 750–900 °C; while the values of *dR/dt* also exhibit linear with dynamic contact angles only at the early stage of reactive spreading at 750–850 °C, or in the whole spreading process at 900 °C. The controlling mechanism of the spreading of Sn-3V alloy on porous graphite cannot be identified from the spreading kinetics data, especially at 900 °C. By estimating the diffusion coefficient of V in liquid Sn as approximately 10^−9^ m^2^/s, the diffusion rate of V atoms in Sn-V droplets can be derived as 10^−4^–10^−5^ m/s [34]. Spreading kinetics indicated the maximum spreading rate of Sn-3V droplets on graphite was around 3.4 × 10^−6^ m/s (see Figure 8d), which is much less than the estimated diffusion rate of V in Sn liquid. Thus, it is can be conceived that, at 750–900 °C, the reactive spreading of Sn-3V alloy should be limited by the kinetics of chemical reactions at the wetting three-phase contact line, which agree with the classic product reaction controlled (PRC) model proposed by Dezellus et al. [33].

**Figure 9:**
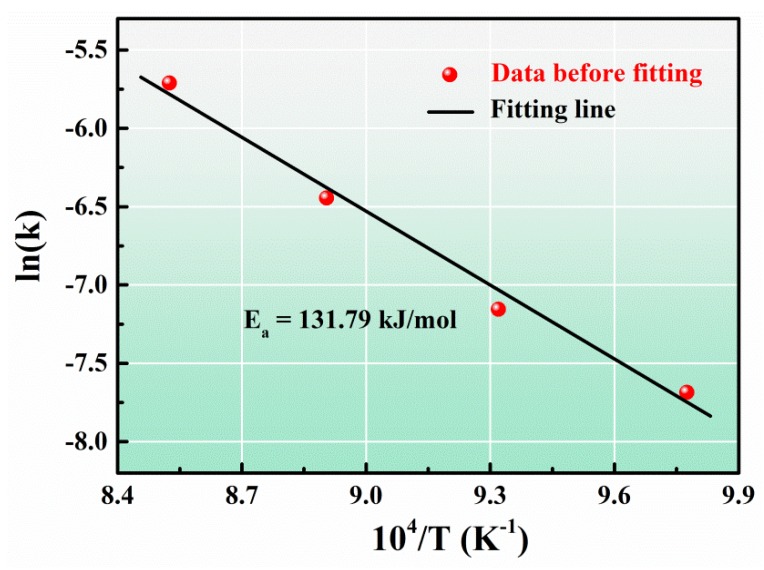
The Arrhenius plotting of kinetic constant k V.S. the reciprocal of isothermal wetting temperature (1/T).

However, vanadium carbides were observed as discontinuous phase behind the advancing three-phase contact line after isothermal wetting at 750–800 °C (see Figure 6a1,b1), which conflicted with the primary assumption of the PRC model of Mortensen et al. [32] and Dezellus et al. [33]. Moreover, as shown in Figure 9, the activation energy of 131.79 kJ.mol^−1^ was calculated by plotting the kinetic constant *k* in Equation (2) against the reciprocal of isothermal wetting temperature using Arrhenius equation [35], which is slightly lower than the typical values of the chemical kinetics limited spreading of Ni-Si alloys [36], Cu-Si and Ni-Si alloys [33] on carbon substrates, but significantly higher than the activation energies of the adsorption-induced spreading of Sn-Ti alloys on alumina ceramics [37]. In particular, the largely negative values of the calculated adsorption energies of V to carbon substrate [27], suggesting that the V atoms released in liquid Sn would be attracted to the Sn-V alloy/porous graphite interface and thus decreased the surface energy at the three-phase contact line of Sn-V alloy molten droplets on porous graphite. Therefore, it is rational to deduce that although they can be described by the classical PRC model, the spreading and infiltrating of Sn-V alloys on porous graphite should be driven by not only the interfacial vanadium formation at the wetting edges (see Figure 6 and Figure 7) but also by the V adsorption to fresh surface of graphite, especially at relatively low temperatures, such as 750 and 800 °C in this work.

### 3.2. Reactive Infiltrating

Driven by the capillary force at the wetting three-phase contact line, liquid metal can wet and spontaneously infiltrate into the pore channels of porous material [38]. Hence, understanding the infiltration of metal in porous graphite is of fundamental importance for the fabrication of graphite compositional materials. Sobczak et al. stated that the infiltration depth of Cu-Ti alloy on porous graphite could reach a few hundred microns, but the infiltration depth of Cu-Cr alloys under the identical condition was negligible in spite of the excellent wetting on porous graphite [39]. This is most likely due to the difference in the nature of the reaction products formed at the drop/substrate interfaces. Lately, Fu et al. reported that the infiltration depth of Sn-Ag-Cu-Ti alloys on porous graphite increased with the increase in Ti content at the constantly elevated temperature up to 1050 °C [21]. According to the theory proposed by Washburn [40], the infiltration depth *h* can be associated with the surface tension *σ*, the viscosity of the liquid metal *η*, the effective pore radius *r*_eff_, the equilibrium contact angle of the liquid on the solid *θ*, and the infiltration time *t* by Equation (3):

(3) $$h^{2}\;=\;r_{\mathrm{eff}}\;\frac{\sigma cos\theta }{2\eta }\;t$$

**Figure 10:**
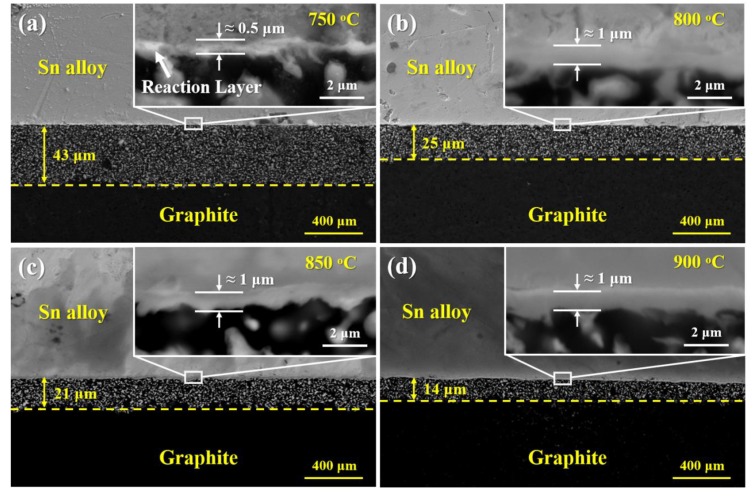
Sn-3V alloy/porous graphite interface microstructures obtained at the center of the solidified Sn-3V alloy droplet after isothermal wetting at (**a**) 750 °C, (**b**) 800 °C, (**c**) 850 °C and (**d**) 900 °C for 3 h.

The prerequisite for the occurrence of infiltration of liquid metal is the equilibrium contact angle *θ* has to be lower than 90°. The infiltration cannot take place in the liquid Sn/porous graphite system due to the high equilibrium contact angle of liquid Sn on porous graphite (around 133° in this work (see Figure 3)). However, the addition of active V element to Sn makes the Sn-V alloy/porous graphite reaction couple a reactive system, in which the equilibrium contact angles on the modified graphite interface are in the range of 11–22° (see Figure 5). As shown in Figure 7, the infiltration process occurred spontaneously in the direction perpendicular to the wetted area nearby three-phase contact line. Figure 10 shows the microstructure underneath the center of the Sn-3V alloy droplets on porous graphite substrate after isothermal wetting at 750, 800, 850, and 900 °C for 3 h. As shown in Figure 10, the infiltration depths of liquid Sn-3V alloys at 750, 800, 850, and 900 °C were 43, 25, 21, and 14 μm, respectively. Furthermore, the thickness of the vanadium carbides layer formed at 750 °C was approximately 0.5 μm (see Figure 10a). As the temperature increased to 800, 850, and 900 °C, the layer thickness of vanadium carbides increased to around 1 μm (see Figure 10b–d). Singh et al. [41] assumed the infiltration of porous carbon in a reactive system was only dependent on the viscosity of liquid metals. However, this assumption is clearly unreasonable and difficult to explain in the light of the experimental results obtain in this work. Voytovych et al. verified that the reactive infiltration in porous graphite was not limited by the viscous flow, but limited by the chemical reaction at the infiltration front of the Ni-Si alloy along with the pores in porous graphite [42]. On the premise that the closure of pores does not occur, the vertical infiltration kinetics in a reactive system is similar to the horizontal spreading kinetics.

In this study, however, the infiltration of Sn-V alloy in the direction perpendicular to the porous graphite surface should be influenced by both the formation of vanadium carbides and the adsorption of active V element at the porous graphite front. When the wettability of the liquid metal on the new compound at the interface is better than that of the initial substrate, the interface product of the new compound can improve the wettability [43]. The vanadium carbides formation would facilitate the infiltration by providing capillary force at infiltrating front; on the other hand, carbides formation in pores also decreased their effective radius [41] and ultimately resulted in the closure of pores, which would completely stop the infiltration of Sn-V alloy in graphite substrate, as shown in Figure 10. It is well known that the growth kinetics of interfacial vanadium carbides is strongly affected by wetting temperature. When wetting reaction temperatures were increased, the growth of interfacial vanadium carbides was accelerated, and hence the closure of the pores in graphite was faster at an elevated wetting temperature. Thus, the rapid formation of the vanadium carbides layer led to a shorter infiltration depth, as observed in this study.

### 3.3. Effects of Porosity

**Figure 11:**
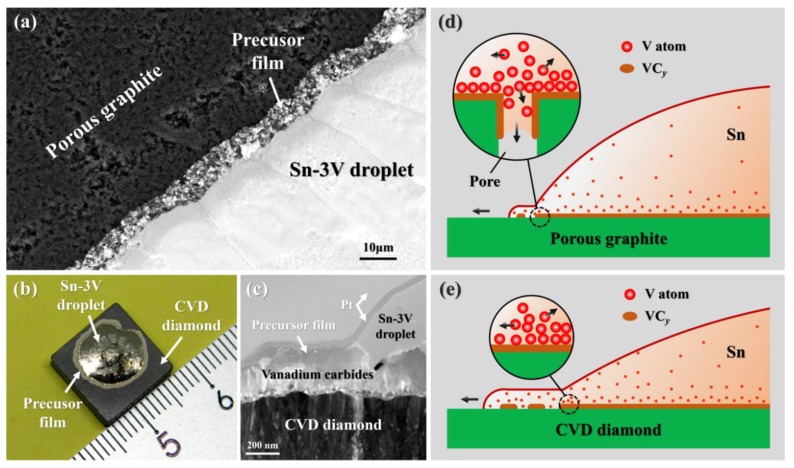
(**a**) top-view SEM micrograph near to the wetting edge of Sn-3V alloy on porous graphite after wetting at 950 °C, (**b**) appearance of Sn-3V alloy/CVD diamond sample wetted at 950 °C, (**c**) bright field TEM image of cross-sectional microstructure at the three-phase contact line of Sn-3V/CVD diamond wetted at 950 °C. The schematic illustration of mass transfer at the triple lines of Sn-3V alloy on (**d**) porous graphite and (**e**) CVD diamond.

The formation of precursor film was commonly observed during the reactive wetting of liquid metals on solid substrates, e.g., Zr-based alloys on Al_2_O_3_ [44] or ZrC [45] substrates, and Sn-Ti alloys/sialon ceramic [46]. However, the precursor film of Sn-V alloys on porous graphite was not obvious, as shown in Figure 2, which is very different from the phenomenon observed on polycrystalline CVD diamond under the same wetting conditions [27]. As shown in Figure 11a, the precursor film formed at the periphery of solidified Sn-3V droplets on porous graphite after wetting at 950 °C was only ~3μm in width. But the width of the precursor film at the triple line of Sn-3V alloy on a polycrystalline CVD diamond substrate for the same experimental conditions reached more than 500 μm, as shown in Figure 11b. Figure 11c shows the cross-sectional TEM bright field image of the microstructure at the three-phase contact line of the corresponding wetting sample in Figure 11b. Clearly, vanadium carbides grew outside the Sn-V droplet, formed part of the precursor film. Through EDS analysis, no matter on porous graphite or polycrystalline CVD diamond substrates, the precursor film mainly consisted of vanadium carbides with some Sn islands formed on its top. According to our previous work [27], formation of the precursor films on carbon materials could be well explained by the model proposed by Xian et al., i.e., the active atoms quickly move to the liquid/solid interface under the attractive force, and then the thin liquid layer enriched by active atoms overflows to the unwetted substrate surface [46]. According to the model suggested by Xian, the accumulated and surplus active atoms at the liquid/solid interface may either diffuse back to the liquid metal, or transfer in front of the wetting three-phase contact line of the spreading droplet under the adsorptive force from the fresh solid surface, as shown in Figure 11e. However, for porous graphite, the pores in the vertical direction would be additional way to eliminate the dynamic stacking of the active atoms at liquid/solid interface (see Figure 11d), which explained why the precursor films were formed more easily on the CVD diamond substrate than porous graphite.

**Figure 12:**
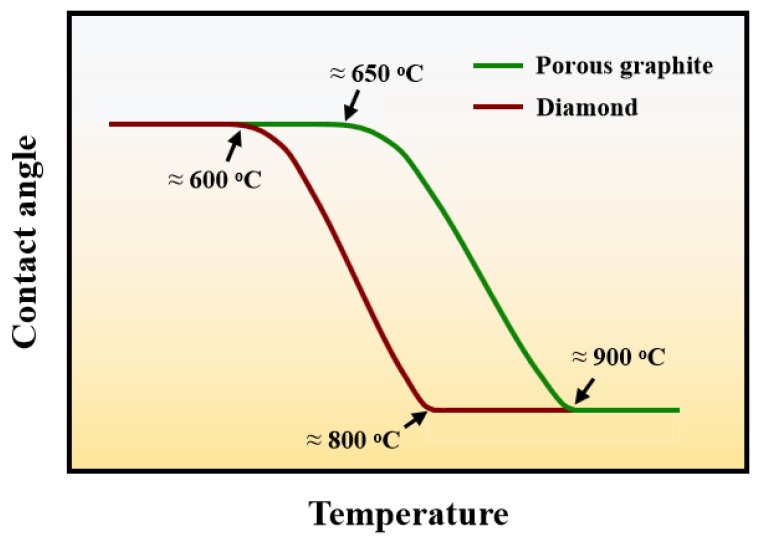
Apparent contact angles of Sn-V alloys on porous graphite and CVD diamond plotted at constantly elevated temperatures up to 950 °C.

The surface morphology of the substrate has an important influence on the apparent contact angles and the precursor film [47], so the wetting behavior of liquid metal on porous graphite and diamond was different. Enhanced precursor films formed on a diamond substrate indicated a better wettability of Sn-V alloys on the diamond than porous graphite, which was experimentally verified [27]. Figure 12 illustrates the typical wettability curves of carbon material at constantly elevated temperatures. The initial temperature for Sn-V alloys to spread on porous graphite is approximately 650 °C, which is 50 °C higher than that on polycrystalline CVD diamond substrate. Furthermore, the spreading of Sn-V alloys on porous graphite reached a quasi-equilibrium state at approximately 900 °C, which is 100 °C higher than that for polycrystalline CVD diamond substrate. Based on above analysis, the difference in the wettability of Sn-V alloy on porous graphite and polycrystalline CVD diamond substrate can be attributed to the difference in patterns of mass transfer: the vertical infiltration process of Sn-V alloys impeded stacking of active V atoms at the liquid/solid interface and hence reduced the spreading kinetics of Sn-V alloys on porous graphite.

## 4. Conclusions

In this work, the reactive spreading and infiltrating of a novel Sn-V alloy on porous graphite were systematically investigated. The conclusions can be drawn based on experimental results and thermodynamic analysis as below:

(1) V concentrations have a minor influence on the final apparent contact angles of Sn-V alloys on porous graphite and a trace doping of 0.5 wt.% V obviously improved the wettability of liquid Sn on porous graphite.
(2) Sn-V alloys approximately started to spread on porous graphite at 650 °C and reached the quasi-equilibrium state at 900 °C. The spreading kinetics of Sn-V alloys on porous graphite at 750–900 °C was well described by the classical chemical reaction-controlled model. However, thermodynamic analysis and associated microstructural characterization evidenced that, besides the formation of vanadium carbides, the adsorption of active V element at the three-phase contact line considerably contributed to the spreading and infiltrating of Sn-V alloys on porous graphite.
(3) The formation of continuous phase of vanadium carbides resulted in the closure of pores, and hence stopped the infiltration of Sn-V alloys in porous graphite substrate. Consequently, the infiltration depth of Sn-V alloys in porous graphite decreased by the accelerated carbides formation at increased wetting temperature.
(4) The difference in mass transfer at the three-phase contact line was accountable for the difference in wetting behaviors between porous graphite and CVD diamond. The presence of pores in graphite substrate impeded the stacking of active V atoms at the wetting three-phase contact line, which was responsible for the difference in the wettability of Sn-V alloy on porous graphite and polycrystalline CVD diamond.

## Supplementary Materials

The following are available online at https://www.mdpi.com/1996-1944/13/7/1532/s1, Figure S1: The cross-sectional microstructure of Sn-1/3/5 alloy/graphite samples cooling from 950 °C, Figure S2: Cross-sectional SEM images of the reaction interfaces at the center of droplet of (a) Sn-0.5V, (b) Sn-1V, (c) Sn-3V, (d) Sn-5V, and (e) Sn-7V, Figure S3: XRD patterns obtained at the interface between Sn-0.5/3/7V alloy and graphite after wetting at 950 °C, inserted with the top-view of Sn-3V/graphite wetting sample after deeply etching, Fugure S4: XRD patterns obtained at the reaction interfaces between Sn-3V alloy and graphite after wetting at 750, 800, 850 and 900 °C. The residual Sn-V alloys at the reaction interface were removed by an HCl solution.
