# Supplementary material for: Reactive Infiltration and Microstructural Characteristics of Sn-V Active Solder Alloys on Porous Graphite
Source: Materials (Basel). 2020 Mar 27;13(7):1532. doi: 10.3390/ma13071532 (PMC7178118; doi:10.3390/ma13071532)
Supplement: Supplementary file 1 [file materials-13-01532-s001.pdf]

Supplementary Materials

Reactive Infiltration and Microstructural Characteristics of Sn-V Active Solder Alloys on Porous Graphite

Yubin Zhang [1,§], Xinjiang Liao [1,§], Qiaoli Lin [2], Dekui Mu [1,*], Jing Lu [1], Hui Huang [1] and Han Huang [3]

1. Institute of Manufacturing Engineering, Huaqiao University, Xiamen 361021, China; 15734003989@163.com (Y.Z.); xinjiangliao@sina.cn (X.L.); lujing26@hqu.edu.cn (J.L.); huangh@hqu.edu.cn (H.H.)
2. School of Materials Science and Engineering, Lanzhou University of Technology, Lanzhou 730000, China; linqiaoli@foxmial.com
3. School of Mechanical and Mining Engineering, The University of Queensland, QLD 4072, Australia; han.huang@uq.edu.au

§ Contributed equally

* Correspondence: dekui.mu@hqu.edu.au; Tel: +86 592 6162616; Fax: +86 592 6162359

Received: 3 March 2020; Accepted: 15 March 2020; Published: date

1. Characterization of Solidified Sn-V Alloys

Figure S1 shows the cross-sectional microstructure of Sn-1/3/5V alloys/graphite wetting samples solidified from 950 °C. Viewing from the optical and SEM images, V and Sn formed Sn-V intermetallics (IMCs) in the droplet during wetting and the amount of Sn-V IMCs increased with the increase of V content. The EDS analysis and Sn-V phase diagram showed that the Sn-V IMCs in the solidified Sn droplet was mainly composed of VSn_2. The refractory VSn_2 formed in the center of Sn droplet would impede the fluidity of Sn-V alloy to a certain extent and result in the formation of a flat platform at the top of Sn-V alloy under the condition of high V content.

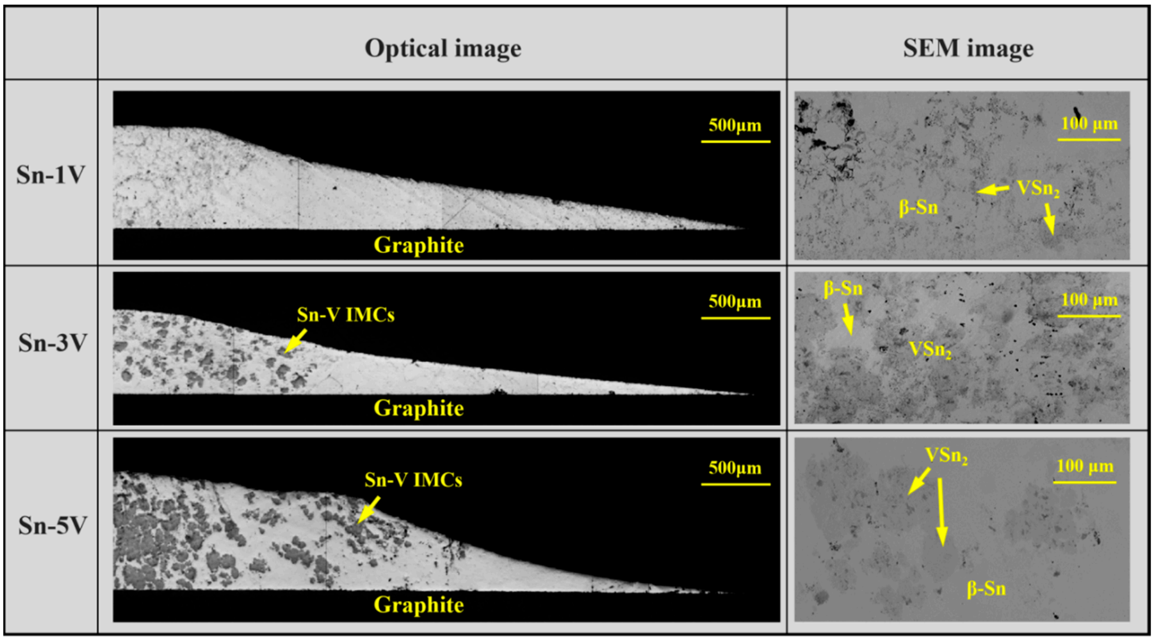

Figure S1. The cross-sectional microstructure of Sn-1/3/5 alloy/graphite samples cooling from 950 °C.

2. Characterization of Reaction Product at Sn-V Alloys/Graphite Interface

Figure S2 shows the cross-sectional microstructure of the reaction interfaces at the center of solidified Sn-V alloys after wetting at 950 °C. EDS analysis suggested that the vanadium carbides

layer was formed adhering to the graphite, which was a barrier for the infiltration of liquid Sn. Moreover, when the V content increased to 3–7 wt.%, a layer of V_3Sn was formed adhering to the vanadium carbides.

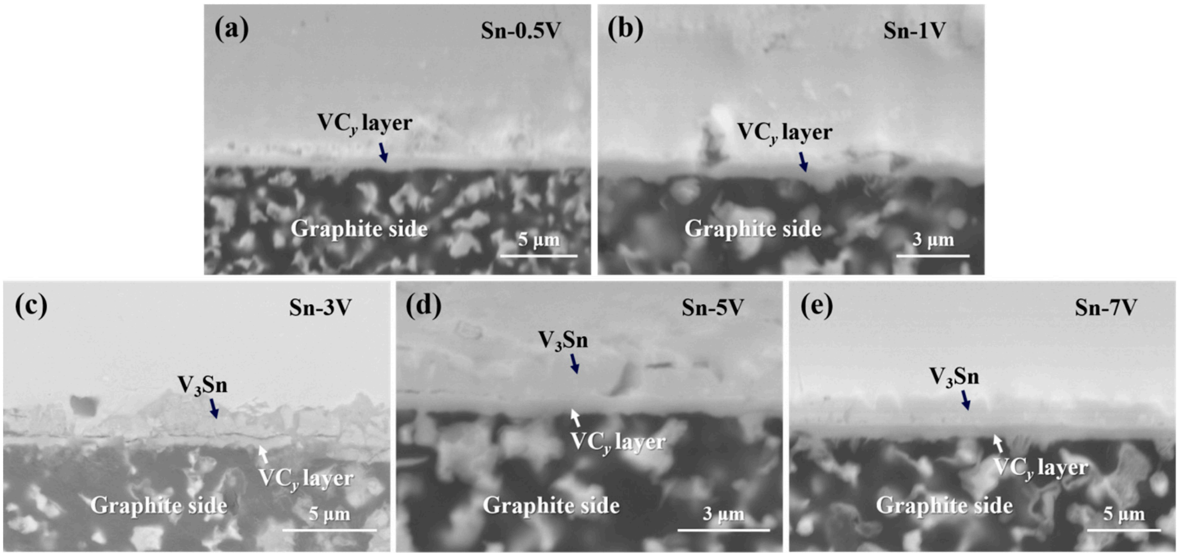

Figure S2. Cross-sectional SEM images of the reaction interfaces at the center of droplet of (**a**) Sn-0.5V, (**b**) Sn-1V, (**c**) Sn-3V, (**d**) Sn-5V, and (**e**) Sn-7V.

Figure S3 shows the XRD patterns acquired at the reaction interfaces between Sn-0.5/3/7V alloys and graphite after wetting at peak temperature of 950 °C. By matching the peak positions with the standard XRD spectra, V_2C, V_8C_5 or V_6C_5 peaks are detected, which indicated the reaction layer was composed of V_2C, V_8C_5 or V_6C_5. Furthermore, the original V content had a limited influence on the composition of the reaction layer.

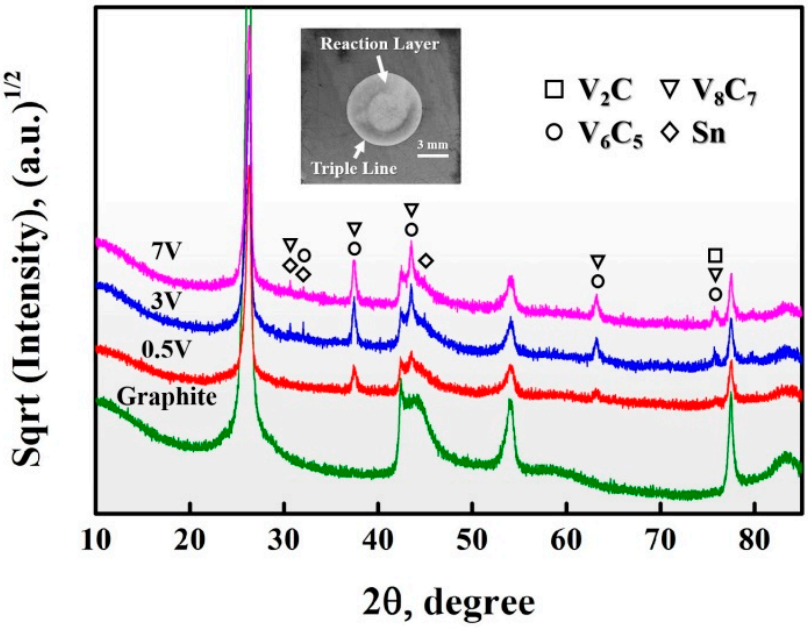

Figure S3. XRD patterns obtained at the interface between Sn-0.5/3/7V alloy and graphite after wetting at 950 °C, inserted with the top-view of Sn-3V/graphite wetting sample after deeply etching.

Figure S4 shows the XRD patterns acquired at the reaction interfaces between Sn-3V alloy and graphite after isothermal wetting at 750, 800, 850, and 900 °C. Similarly, the reaction layer was composed of V_2C, V_8C_5 or V_6C_5 according to the peak positions shown in Figure S4.

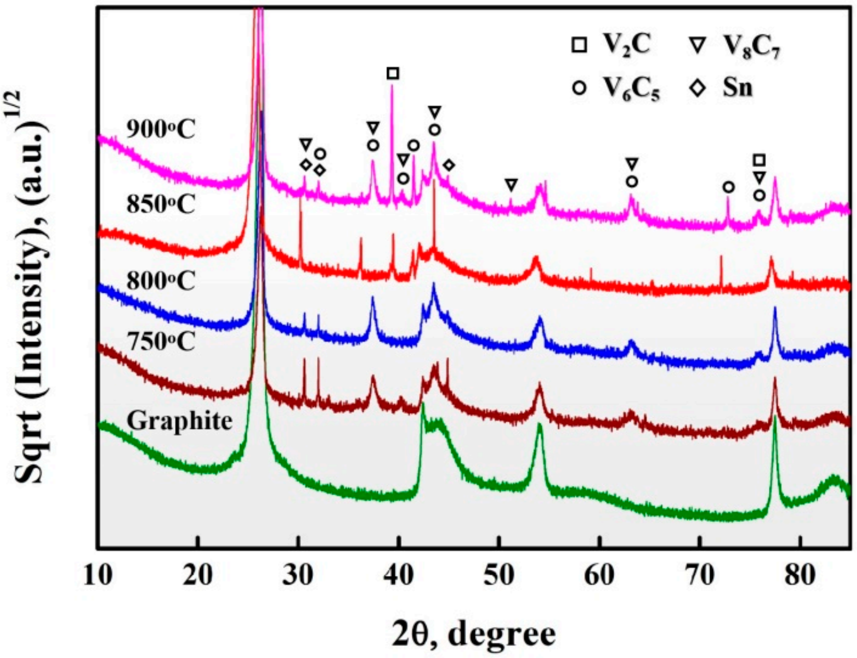

Figure S4. XRD patterns obtained at the reaction interfaces between Sn-3V alloy and graphite after wetting at 750, 800, 850 and 900 °C. The residual Sn-V alloys at the reaction interface were removed by an HCl solution.

© 2020 by the authors. Licensee MDPI, Basel, Switzerland. This article is an open access article distributed under the terms and conditions of the Creative Commons Attribution (CC BY) license (http://creativecommons.org/licenses/by/4.0/).
